# Toward sustainable development goals in gender inequality: an analysis of gender preferences among urban pregnant women in a Southeast Asian country

**DOI:** 10.1186/s12884-023-06109-z

**Published:** 2023-11-10

**Authors:** Anh Duy Nguyen, Long Hoang Nguyen, Lam Duc Nguyen, Ly Thi Ninh, Ha Thu Thi Nguyen, Cuong Tat Nguyen, Nila Nathan, Anh Linh Do, Anh Minh Le, Linh Phuong Doan, Son Hoang Nguyen, Thuc Minh Thi Vu, Bach Xuan Tran, Carl A. Latkin, Cyrus S.H. Ho, Roger C.M. Ho

**Affiliations:** 1 Hanoi Obstetrics and Gynecology Hospital, Hanoi, Vietnam; 2 Department of Global Public Health, Karolinska Institutet, Stockholm, Sweden; 3 Department of Anesthesiology, Hanoi Medical University, Hanoi, Vietnam; 4 Social Affair Department, Ca Mau Obstetrics & Pediatrics Hospital, Ca Mau, Vietnam; 5 Institute for Global Health Innovations, Duy Tan University, Da Nang, 550000 Vietnam; 6 Faculty of Medicine, Duy Tan University, Da Nang, Vietnam; 7 Quantitative Biomedical Sciences, Geisel School of Medicine, Dartmouth College, Hanover, NH USA; 8 Institute of Health Economics and Technology, Hanoi, Vietnam; 9 Center of Excellence in Evidence-based Medicine, Nguyen Tat Thanh University, Ho Chi Minh City, 700000 Vietnam; 10 Institute for Preventive Medicine and Public Health, Hanoi Medical University, Hanoi, Vietnam; 11 Bloomberg School of Public Health, Johns Hopkins University, Baltimore, MD USA; 12 Department of Psychological Medicine, Yong Loo Lin School of Medicine, National University of Singapore, Singapore, Singapore; 13 Institute for Health Innovation and Technology (iHealthtech), National University of Singapore, Singapore, Singapore

**Keywords:** Gender preferences, Pregnant women, Sex selection, Inequality

## Abstract

**Background:**

Gender-biased discrimination and preferences are global phenomena, particularly son preferences. However, updated evidence about this issue in Vietnam has not yet been provided. Therefore, this study aimed to examine the gender preferences among pregnant women and identify associated factors of such preferences.

**Methods:**

A cross-sectional survey was conducted in two hospitals in Vietnam with 732 pregnant women. Gender preferences for their child were asked, along with socio-demographic (e.g., education, occupation) and pregnancy characteristics (e.g., pressure to have a son, gender of first child, the importance to have a son of family members, and information sources on pregnancy care) by using face-to-face interviews and a structured questionnaire. Multinomial logistic regression was performed to determine factors associated with gender preferences.

**Results:**

About 51.9% of the participants had no gender preference, while, among those who had a gender preference, 26.5% preferred sons, and 21.6% preferred daughters. Only 6.2% had pressure to have a son. Having the first child who was female (OR = 4.16, 95%CI = 1.54–11.25), having the pressure to have a son (OR = 6.77, 95%CI = 2.06–22.26), and higher self-perceived importance to have a son (OR = 3.05, 95%CI = 1.85–5.02) were positively associated with son preference. Otherwise, women having partners with high school education or above (OR = 2.04, 95%CI = 1.06–3.91), living with parents-in-law (OR = 2.33; 95%CI = 1.25–4.34), the higher number of pregnancies, and a higher degree of importance in having a son regarding parents-in-law (OR = 2.15, 95%CI = 1.38–3.35) associated with higher odds of preferring daughter.

**Conclusion:**

This study showed that gender preference was common among pregnant women, but the pressure to have a son was low. Further education programs and legal institutions should be implemented to improve gender inequality and gender preference in society.

## Introduction

One of the Sustainable Development Goals (SDG17) is to ensure gender equality and non-gender discrimination at all levels [1]. Nonetheless, gender-biased selection at birth is still a widespread socio-cultural issue in many countries and communities [2, 3]. This is a great issue that can cause gender imbalance and affect the human rights [4]. Since 1994, and more recently, 2011, the United Nations and its affiliated organizations including the Office of the High Commissioner for Human Rights (OHCHR), United Nations Population Fund (UNFPA), United Nations Children’s Fund (UNICEF), United Nations Entity for Gender Equality and the Empowerment of Women (UN Women), and World Health Organization have issued a joint statement affirming the need to eliminate gender-based discrimination, including son preference [4, 5]. Many countries around the world have also introduced gender equality policies and limited sex selection at birth, as well as implemented community interventions to raise awareness of people’s gender equality rights and sex selection [6–8].

In practice, however, sex selection at birth is still prevalent in many countries and cultures across the globe [2, 3]. This phenomenon is especially common in Asian countries such as Korea, China or India, Nepal, and Vietnam, particularly for the first child [9–13]. Countries with large Asian communities also have this situation such as the United States, Canada, or the United Kingdom [14–16]. Traditionally, with male privileges observed in societies [17], it is understandable that couples tend to prefer boys. In East Asian countries like China or Vietnam, which are influenced by Confucianism ideology, the son is associated with the role of the head of the family, and the main person in charge of taking care of his parents and ancestor worship [6, 10]. This has put considerable social and family pressure on women as they need to ensure they will have a son. In recent decades, gendered institutions and gender roles have undergone significant changes, leading to a shift in gender preferences for offspring. Notably, son preference has gradually given way to a distinct daughter preference. Empirical evidence from a nationwide survey in Japan revealed that 75.7% of women, in the event of having a single child, expressed a preference for a female baby [18], representing a considerable departure from a decade ago when only 48.5% of women shared this preference [18]. Meanwhile, in India, there seems to be little disparity in the desire to have a male or female child. According to a Pew Research Center survey conducted from 2019 to 2020, 94% of women consider it “very important” for a family to have at least one son, while 90% hold the same view for having at least one daughter [19]. These results indicate that the vast majority of Indian adults consider both sons and daughters to be integral components of a family. It is noteworthy that daughter preference has emerged despite Japan and India’s more traditional gender relations, which diverge from those of other developed nations.

Although the issue of sex selection at birth is proved to be unethical practice and should be restricted, some argue that there should be no restriction because it is the right to freedom and autonomy of couples in the reproduction [20–22]. The concept of “family balancing” is proposed when couples want sex selection before birth to ensure the gender balance among children in the family [23, 24]. However, regardless of the reasons, gender preference and sex selection can greatly affect the natural gender balance [25]. Therefore, intervention strategies in promoting awareness of gender preference and sex selection restriction still need to be implemented to address this issue.

In Vietnam, there is a gender imbalance occurring in most localities [26], and the sex ratio at birth in Vietnam tends to increase over time, from 1.08:1 (male/female ratio) in 2000–2005 to 1.12:1 in 2015–2020 [27]. Gender preference, particularly, is more common in rural areas than in urban areas [28, 29]. The increase in the male-biased sex ratio is a concern of policymakers. The Decision No. 1679/QD-TTG dated November 22, 2019, of the Prime Minister on the approval of the Vietnam Population Strategy to 2030 [30] or resolution 21-NQ/TW of the Communism Party [31] underlines the gender inequality and pervasiveness of sex imbalance at birth and sets the goal of bringing the sex ratio at birth to the natural equilibrium level. However, with rapid urbanization, economic growth, and the need to reduce the size of households, sex selection among urban couples is common because they want to have children with the desired gender from the first pregnancy. Therefore, it is necessary to have up-to-date evidence on the gender preferences of these women, thereby helping to develop intervention programs to improve this phenomenon and help ensure the quality of the population of Vietnam in the future.

Several previous studies have suggested that age, level of education, socioeconomic status, presence of living children, family members having gender preference as well as issues related to pressure, anxiety, and stress were the main predictors for antenatal gender preference [32–35]. However, currently, the evidence on gender preference in urban pregnant women of Vietnam is still limited. Particularly, studies and evidence on gender disparity, preference for sex selection at birth in Vietnam are largely based on local data, small-scale surveys focusing on abortion, and especially qualitative studies [36–40]. The objective of this study was to investigate the gender preferences of pregnant women and determine the factors that influence their preferences. Specifically, we sought to assess the impact of variables such as education level, social and familial pressure, the gender of their first child, satisfaction with their family, and sources of information on pregnancy care. Based on our findings, we aim to develop and suggest interventions that address these factors effectively.

## Materials and methods

### Study design, sampling method, and data collection

This cross-sectional study was performed at Hanoi Obstetrics and Gynecology Hospital (Northern Vietnam) and Ca Mau Obstetrics and Children’s Hospital (Southern Vietnam) from January to February 2021. We recruited pregnant women aged 18 years or above and voluntarily participated in the study in gave written informed consent. Both pregnant women who had visited hospitals for regular examination or childbirth delivery during the study period were included. We excluded individuals having conditions such as cognitive impairment or other disabilities that might affect their ability to respond to the survey. We used a convenient sampling technique to recruit participants. All eligible pregnant women were approached and briefly introduced to the study. Among 1019 pregnant women who were invited, a total of 732 pregnant women responded to the question about gender preferences (response rate 71.8%). Data from these individuals were finally used for analysis. No difference regarding age, education, occupation, history of pregnancy, and the number of children between pregnant women who were included and excluded. The study was approved by the Institutional Review Boards of Hanoi Obstetrics & Gynecology Hospital (No: 07QĐ/PS-TTĐT).

### Measures and instruments

In this study, the research questionnaire was developed based on a standard procedure. First, we carried out a systematic review to assess the situation and important facets associated with gender preference that have been mentioned in previous studies as well as identify the gaps of issues that needed further studies. Next, a research instrument was developed to cover all aspects of interest. In this process, we invited groups of obstetrics experts, population experts, and policymakers to jointly develop and discuss the content, rephrase, and logical order of the instrument. Before collecting data, the questionnaire was piloted on 10 pregnant women and revised based on their comments to once again ensure the logical order, language, and texts. Finally, a structured questionnaire with five main sections was used in this study, including (1) Socio-demographic characteristics; (2) Pregnancy characteristics, (3) Pressure of pregnant women, (4) Satisfaction of pregnant women with their family’s care; and (5) Childbearing and gender preferences. During the data collection period, if they accepted to be study participants, a face-to-face interview was conducted for 15–20 min by investigators who were well-trained to use questionnaires. The interview took place in a closed room, to ensure privacy and limit outside influences. Collected data was saved in a secured system and only served for study purposes. Variables of interest included:

## Primary outcomes

### Childbearing and gender preferences

In this study, to assess gender preference among pregnant women, we asked participants a question “In this pregnancy, does the sex of your baby matter to you? If yes, would you prefer your child this time to be male or female?” with three answers options, including son preference, daughter preference, and no gender preference.

### Covariates

#### Socio-demographic characteristics

Information about region (North / South), living location (rural areas / urban areas), level of pregnacy’s education ( below high school / high school / colleges / university or above), partner’s education ( below high school / high school / colleges / university or above), occupation (farmer, blue-collar worker / public servant / office worker / housewife / others), having health insurance (yes / no), age (years), and partner’s age (years), living arrangements (parents in law / parents), and monthly household income (VND) was collected. In terms of occupation variable, we based on the definition of the International Labour Organization to categorize some types of occupation into groups. For example, blue-collar workers perform manual work, including persons who are skilled in various trades (carpenters, welders, construction workers, foremen, operators of certain types of equipment, motor vehicle drives) as well as unskilled or semi-skilled and maintenance workers. They are also often traditionally paid on a weekly, and hourly [41]. The public service personnel comprise persons employed by public authorities at central, regional and local levels and include both civil servants and public employees [42]. Monthly household income was also exchanged from VND to US$ (January 2021 exchange rate [43]).

#### Pregnancy characteristics

We asked pregnant women to report their number of pregnancies, gender of the first child, frequency of antenatal care, preferable delivery method, having any pregnancy complications during the pregnancy period, and information sources on pregnancy care.

#### The pressure of pregnant women

Information about the pressure to have a son, and desired number of children were collected. Furthermore, we also asked participants to rate the importance to have a son with themselves, their partners, parents-in-law, and their parents on a 5-point Likert scale from 1 “Completely not important” to 5 “Completely important”.

#### Satisfaction of pregnant women with their family’s care

We examined the level of satisfaction of pregnant women with their husband/partner, parents-in-law, and parents on an 11-point Likert scale from 0 “Complete dissatisfaction” to 10 “Complete satisfaction”.

### Statistical analysis

Data were analyzed by Stata software version 15.0 (Stata Corp. LP, College Station, TX, USA). A p-value < 0.05 was considered statistically significant. Descriptive statistics were calculated to compare the gender preferences in different socio-demographic characteristics, history of maternity care, and childbearing. Chi-squared and Kruskal-Wallis tests were performed to examine the differences. We used multinomial logistic regression model for identifying factors associated with gender preferences (desire to have a son/daughter among pregnant women versus no preference). In this study, the regression model was also adjusted by some potential variables. Particularly, variables that were put on the full regression models included socioeconomic status, history of maternity care, and childbearing characteristics. The forward stepwise approach was utilized to develop the reduced regression model, with a p-value of < 0.02 as a threshold to include variables in the model.

## Results

The socio-demographic characteristics of participants are described in Table 1. There was a difference in gender preference among participants. Particularly, about 51.9% of the participants had no gender preference, while, among those who had a gender preference, 26.5% preferred sons, and 21.6% preferred daughters. Only 6.2% had pressure to have a son. In terms of the differences between gender preference and some socio-demographic characteristics, firstly, participants in South areas had a significantly higher proportion of gender preference than North (son: 36.7% vs. 23.5%; daughter: 28.4% vs. 19.6%; p < 0.01). In terms of the levels of education, people below high school level had a dramatically higher preference for gender preference, with 43.8% and 24.7% of them preferring son and daughter, respectively. The difference in gender preference between levels of education was statistically significant with p < 0.01. People who were farmers, or blue workers also reported a higher level of gender preference compared to other occupations, with 34.0% and 22.0% of them preferred for son and daughter, respectively. The difference in gender preference between types of occupations was statistically significant with p = 0.03.

**Table 1:** Socio-demographic of participants

Characteristics	Total		Son preference		No preference		Daughter preference		p
	n	%	n	%	n	%	n	%	
**Total**	732	100.0	194	26.5	380	51.9	158	21.6	
**Region**									
North	561	76.9	132	23.5	319	56.9	110	19.6	< 0.01
South	169	23.2	62	36.7	59	34.9	48	28.4	
**Living location**									
Rural	252	34.4	78	31.0	113	44.8	61	24.2	0.02
Urban	480	65.6	116	24.2	267	55.6	97	20.2	
**Education**									
< High school	89	12.2	39	43.8	28	31.5	22	24.7	< 0.01
High school	167	22.9	52	31.1	75	44.9	40	24.0	
Colleges	144	19.7	39	27.1	77	53.5	28	19.4	
University or above	331	45.3	64	19.3	199	60.1	68	20.5	
**Partner’s education**									
< High school	63	8.6	21	33.3	24	38.1	18	28.6	< 0.01
High school	146	20.1	41	28.1	76	52.1	29	19.9	
Colleges	200	27.4	62	31.0	83	41.5	55	27.5	
University or above	320	43.9	70	21.9	195	60.9	55	17.2	
**Occupation**									
Farmer, blue-collar worker	100	13.7	34	34.0	44	44.0	22	22.0	0.03
Public servant	62	8.5	15	24.2	33	53.2	14	22.6	
Office worker	292	39.9	65	22.3	169	57.9	58	19.9	
Housewife	165	22.5	57	34.5	71	43.0	37	22.4	
Others	113	15.4	23	20.4	63	55.8	27	23.9	
**Having insurance**	483	66.0	108	22.4	284	58.8	91	18.8	< 0.01
**Living arrangements**									
Parents in law	311	42.5	85	27.3	153	49.2	73	23.5	0.41
Parents	67	9.2	20	29.9	32	47.8	15	22.4	0.75
	**Mean**	**SD**	**Mean**	**SD**	**Mean**	**SD**	**Mean**	**SD**	
**Age**	29.4	5.0	29.6	5.2	29.2	4.9	29.7	5.0	0.33
**Partner’s age**	32.6	5.5	32.8	5.7	32.1	5.6	33.2	5.2	0.02
**Monthly household income** (Unit:USD)	682.1	542.2	633.3	507.8	716	568.7	647.6	504.1	0.21

Table 2 presents the history of maternity care and satisfaction with care from family among pregnant women. The results showed that the proportion of women who visited the hospital more than one month per visit having no gender preference (41.3%) was the lowest compared to other groups (p = 0.02). Individuals who sought information from Friends/Relatives, Internet/social media, Radio/TV, Newspapers/books, and Smartphone applications had a higher proportion of having no gender preference in comparison with those without these sources (p < 0.05). Table 2 also indicates that women with no preference had significantly higher levels of satisfaction with their husbands/partners, parents-in-law, and parents compared to those with specific gender preferences (p < 0.05).

**Table 2:** History of maternity care and satisfaction of pregnant women with their family’s care

Characteristics	Total		Son preference		No preference		Daughter preference		p
	n	%	n	%	n	%	n	%	
**Frequency antenatal care**									
Weekly	84	11.5	22	26.2	45	53.6	17	20.2	0.02
Monthly	238	32.5	50	21.0	145	60.9	43	18.1	
More than a month	121	16.5	40	33.1	50	41.3	31	25.6	
Follow your physician’s instructions	289	39.5	82	28.4	140	48.4	67	23.2	
**Preferable delivery method**									
Vaginal delivery	326	49.8	89	27.3	174	53.4	63	19.3	0.34
Caesarean delivery	328	50.2	90	27.4	160	48.8	78	23.8	
**Having any pregnancy’ complications**	177	24.2	48	27.1	88	49.7	41	23.2	0.77
**Information sources on pregnancy care**									
Friends / relatives	365	49.9	75	20.5	222	60.8	68	18.5	< 0.01
Posters/Banners	25	3.4	5	20.0	16	64.0	4	16.0	0.47
Internet/Social media	511	69.8	110	21.5	303	59.3	98	19.2	< 0.01
SMS/Message	48	6.6	11	22.9	31	64.6	6	12.5	0.15
Radio/Television	132	18.0	21	15.9	95	72.0	16	12.1	< 0.01
Newspapers/Book	139	19.0	29	20.9	88	63.3	22	15.8	0.01
Health worker	393	53.7	105	26.7	196	49.9	92	23.4	0.37
Smartphone app	162	22.1	27	16.7	111	68.5	24	14.8	< 0.01
	**Mean**	**SD**	**Mean**	**SD**	**Mean**	**SD**	**Mean**	**SD**	
**Satisfaction with care** **(0–10)**									
Husband/ Partner	8.6	2.1	8.1	2.1	9.0	1.8	8.0	2.3	< 0.01
Parents-in-law	8.2	2.4	7.7	2.4	8.7	2.2	7.6	2.6	< 0.01
Parent	8.8	2.0	8.2	2.2	9.2	1.6	8.4	2.1	< 0.01

Characteristics of childbearing, and the desire to have children of respondents are described in Table 3. Most of the participants had one time of pregnancy (44.7%), their first child was male (44.7%) and the majority of them preferred to have two children (67.6%). Most of the participants did not have pressure to have a son (93.9%). Pregnant women who preferred a son rated a higher level of importance to have a son regarding themselves, husband, parents-in-law, and parents compared to those preferring a daughter and those having no preference (p < 0.05).

**Table 3:** Childbearing, the desire to have children of respondents

Characteristics	Total		Son preference		No preference		Daughter preference		p
	n	%	n	%	n	%	n	%	
**Number of pregnancies**									
1 time	324	44.7	88	27.2	192	59.3	44	13.6	< 0.01
2 times	278	38.3	62	22.3	132	47.5	84	30.2	
3 times and above	123	17.0	42	34.1	53	43.1	28	22.8	
**Gender of first child**									
None/Unknown	133	18.2	19	14.3	91	68.4	23	17.3	< 0.01
Male	327	44.7	82	25.1	141	43.1	104	31.8	
Female	271	37.1	93	34.3	147	54.2	31	11.4	
**Desired number of children**									
One	14	2.2	4	28.6	6	42.9	4	28.6	< 0.01
Two	422	67.6	77	18.2	258	61.1	87	20.6	
3 children and above	188	30.1	62	33.0	93	49.5	33	17.6	
**The pressure to have a son**									
No	687	93.9	167	24.3	371	54.0	149	21.7	< 0.01
Yes	45	6.2	27	60.0	9	20.0	9	20.0	
	**Mean**	**SD**	**Mean**	**SD**	**Mean**	**SD**	**Mean**	**SD**	
**The importance to have son (1–5)**									
Self	2.3	0.9	2.8	0.8	2.1	0.8	2.1	0.8	< 0.01
Husband/ Partner	2.5	1.0	3.0	0.9	2.3	0.9	2.3	1.0	< 0.01
Parents-in-law	2.7	1.0	3.1	0.9	2.5	1.0	2.6	1.1	< 0.01
Parents	2.4	0.9	2.8	0.9	2.2	0.9	2.2	0.9	< 0.01

Table 4 shows factors associated with gender preferences among pregnant women. High school education or above (OR = 0.32, 95%CI = 0.16–0.65) and using smartphone applications as an information source (OR = 0.48, 95%CI = 0.24–0.99) were negatively associated with son preference. Meanwhile, having the first child who was female (OR = 4.16, 95%CI = 1.54–11.25), having the pressure to have a son (OR = 6.77, 95%CI = 2.06–22.26), and higher self-perceived importance to have a son (OR = 3.05, 95%CI = 1.85–5.02) were positively associated with son preference.

**Table 4:** Multinomial logistic regression identified factors associated with gender preferences among pregnant women

Characteristics	Son preference		Daughter preference	
	OR	95%CI	OR	95%CI
**INDIVIDUAL CHARACTERISTICS**				
**Education** (High school or above vs. < High school)	0.32***	0.16; 0.65	0.81	0.39; 1.69
**Education of Partne**r (High school or above vs. < High school)	1.31	0.69; 2.46	2.04**	1.06; 3.91
**Occupation** (vs. Farmer, worker-ref)				
Office worker	1.38	0.67; 2.85	2.07*	0.93; 4.61
Others	0.88	0.37; 2.07	3.16***	1.32; 7.57
**Living with parents-in-law (Yes vs. No-ref)**	1.02	0.57; 1.82	2.33***	1.25; 4.34
**Having insurance (Yes vs. No-ref)**	0.70	0.34; 1.43	0.43**	0.20; 0.92
**Husband’s age**	1.03	0.98; 1.09	0.95	0.89; 1.02
**Monthly household income**	1.00	1.00; 1.00	1.00	1.00; 1.00
**MATERNITY CARE**				
**Number of pregnancies** (vs. Once-ref)				
2 times	0.88	0.43; 1.83	11.55***	4.61; 28.91
3 times and above	1.46	0.57; 3.71	19.58***	5.86; 65.38
**Gender of first child** (vs. None/Unknown - ref)				
Male	2.52*	0.96; 6.60	0.77	0.30; 1.97
Female	4.16***	1.54; 11.25	0.08***	0.02; 0.24
**Pressure to have a son** (Yes vs. No-ref)	6.77***	2.06; 22.26	2.84	0.64; 12.60
**The importance to have a son**				
Self (per score)	3.05***	1.85; 5.02	0.67	0.40; 1.13
Parents-in-law (per score)	1.11	0.69; 1.77	2.15***	1.38; 3.35
Parent (per score)	1.25	0.78; 2.02	0.48***	0.29; 0.79
**Satisfaction**				
Parents-in-law (per score)	0.96	0.82; 1.12	0.82**	0.71; 0.96
Parent (per score)	0.85	0.70; 1.03	1.10	0.89; 1.35
**Information sources on pregnancy care**				
Radio/Television (Yes vs. No - ref)	0.58	0.26; 1.28	0.20***	0.08; 0.52
Health worker (Yes vs. No - ref)	0.77	0.42; 1.42	1.93*	0.99; 3.78
Smartphone app (Yes vs. No - ref)	0.48**	0.24; 0.99	0.59	0.28; 1.24

*** p < 0.01, ** p < 0.05, * p < 0.1

In terms of daughter preference, women having partners with high school education or above (OR = 2.04, 95%CI = 1.06–3.91), living with parents-in-law (OR = 2.33; 95%CI = 1.25–4.34), the higher number of pregnancies, and a higher degree of importance in having a son regarding parents-in-law (OR = 2.15, 95%CI = 1.38–3.35) associated with higher odds of preferring daughter. Otherwise, daughter preference was negatively associated with having insurance (OR = 0.43, 95%CI = 0.20–0.92), having the first child who was female (OR = 0.08, 95%CI = 0.02–0.24), the degree of importance in having a son regarding parents of pregnant women (OR = 0.48, 95%CI = 0.29–0.79), the degree of satisfaction with parents-in-law’s care (OR = 0.82, 95%CI = 0.71–0.96), and using Radio/Television as an information source (OR = 0.20, 95%CI = 0.08–0.52).

## Discussion

This study contributed to the body of literature about the progress of gender equality regarding the improvement of sex selection at birth and gender preference in Vietnam. The results showed that above a half of the women in the study had no specific gender preferences and most of the participants also did not have any pressure to have a son. Factors related to gender preferences through a multivariable regression model also suggest potential implications in improving this phenomenon in Vietnam.

The current study revealed a son preference of 26.5%, which exceeded the daughter preference of 21.6%. These findings are consistent with several previous studies. For instance, Kumar Nithin et al. reported son preference at 22.0%, while daughter preference was only 17.4% [44]. Similarly, Thakkar et al. found that 22.2% of women desired a male child, compared to 14.4% who preferred a female child [45]. Karmali et al. conducted a study in Goa that demonstrated 23.1% of women had a preference for a male child [46]. The issue of gender disparity at birth is widely recognized as a demographic problem in numerous countries and cultures [2, 3]. Since the development of ultrasound and pre-pregnancy sex selection technologies, some countries, such as China, Vietnam, India, and Nepal have shown an increasingly different male/female ratio [47–50]. The belief in these countries is that men are better at managing and shouldering family matters than women, that they will help their parents when they are old, and they will continue running the family business. Even in some countries, daughters are considered an economic burden because they must have a dowry when their daughter returns to her husband’s house and stays at her husband’s house after marriage [51]. On the other hand, due to the population structure and the quality of the system changing, people are afraid of having too many children and want the current child to be born to be a boy to fulfill the wife’s obligations. To be able to choose the current gender, many people have colluded with health officials to have access to ultrasound and abortion services to determine gender and select sex. [52, 53]. The Vietnam government’s response to the phenomenon of gender imbalance and gender selection through policies prohibiting the selection of the sex of the fetus through health education and communication, diagnosing the fetus, or excluding the fetus for the reason of sex selection [54]. However, it does not seem to have been widely applied, as the rate of imbalanced sex selection is still taking place in most provinces [26]. Despite the disparity in the desire to have a male or female child, it is reassuring to observe that 51.9% of pregnant women in the present study did not exhibit any particular gender preference. This finding aligns with the results of several earlier studies [44, 55, 56]. These outcomes are encouraging and represent a positive step towards achieving goal 3 of the millennium development goals. Nevertheless, more rigorous policies are necessary to prevent sex selection practices. Persistent efforts are required to improve the current scenario.

The results showed that the rate of pressure to have a son was low, but it is reaffirmed as a major factor affecting the son preference. Vietnamese women are often pressured by their husbands and husbands’ family to give birth to a son. A prior qualitative study showed that women were often pressured to have children and wanted to have a son [39]. Women often use three basic strategies to negotiate the need to have a son: (1) to have as many children as possible until a boy is born; (2) to find a second wife for her husband and (3) to adopt a son. Under pressure from many sides, they are determined to have a son. Community influence is the largest agent in shaping reproductive desires and behavior. Women have difficulty facing the pressure and they end up breaking the two-child policy to have a boy [39]. Therefore, it is necessary to have sanctions and laws to prohibit the act of sex selection. In Europe, Article 14 of the European Convention on Human Rights and Biomedical Medicine (Oviedo Convention) of 1997 states that medically assisted reproductive techniques are not be permitted to select the sex of a child in the future, except in severe cases where the associated disease must be avoided [57]. Some countries like Austria and Switzerland forbid selection for any reason [57]. A good finding in this study is that the rate of pressure to have a son was low. In addition, the findings showed that the importance of having a son for pregnant women affected the desire to have a son. This is because currently women have more autonomy and rights as opposed to the past and are not under the influence of outside pressure such as her family or her husband. The desire to have a son only comes from themselves in this case.

Mothers who already had a daughter had no or less desire for a daughter than mothers who had given birth for the first time. Specifically, those who have had their first child, a girl, were more likely to have a son than those who have never had a child. This result is consistent with the context in Vietnam, where the desire to have a son is high. When the first child is a girl, there is a need for the next child to be a boy to have both men and women. This result is consistent with the study of Yamaguchi, K [58, 59]. This finding was also in line with the trend of the family balancing [23, 24]. The results also showed a relationship between mothers’ education level and sex selection, which was similar to the previous findings [60]. We found that people with a lower education level have a higher preference for sons than those with a higher level of education. These results are entirely consistent with the findings of the International Center for Research on Women (ICRW), which indicated that maternal education is among the most crucial factors in reducing son preference [44]. This can be explained by the fact that highly educated individuals possess a greater level of health literacy and an open-minded approach to a daughter’s role in the family. They tend to be less concerned about the sex of their children and prioritize ensuring that their child is appropriately cared for and develops optimally.

Currently, with the development of information technology and the explosion of telemedicine, information can be obtained instantaneously. Through smartphone applications, pregnant women can interact with their physicians remotely and timely. Now the information is easily obtained, so the child’s gender knowledge is also updated, less dependent on the medical staff or have interaction without having to come in person [61]. Our results are in line with current trends. Thanks to searching for information on maternity care, those who use apps have a lower desire to have a boy than those who don’t use smartphone apps. The reason for this phenomenon is not clear; however, we assumed that mothers who used smartphone applications often have high levels of education, and therefore are less concerned about the gender of their children as discussed above.

The findings of this study provide several recommendations to enhance policy. First, educational campaigns should be performed to raise awareness of sex selection and gender preferences, particularly among individuals with low levels of education and their families, with the goal of altering their attitudes and behaviors. Second, legal institutions should be strengthened by rigorously enforcing laws against noncompliance, especially in cases of sex-selective abortions. Finally, comprehensive action plans aimed at reducing gender-based discrimination in society and empowering women in the community should be implemented effectively, which could ultimately lead to a decrease in gender preferences for children.

There are some limitations in this study. First, since it is a cross-sectional study, the cause-and-effect relationship has not been shown, and further research is needed to cover the issue as a longitudinal follow-up study. Second, the convenience sampling at the two hospitals did not cover all populations in Vietnam. For example, we were unable to have data on the central region of Vietnam. Therefore, more studies on a larger scale, over a longer period with diverse contexts are needed to identify the socio-economic factors affecting sex selection.

## Conclusion

To conclude, this study showed that gender preference was common among pregnant women in our settings, but the pressure to have a son was low. Further education programs and legal institutions should be implemented to improve gender inequality and gender preference in society.

## Data Availability

The data that support the findings of this study are available from the Hanoi Medical University but restrictions apply to the availability of these data, which were used under license for the current study, and so are not publicly available. Data are however available from the authors upon reasonable request and with permission of Hanoi Medical University (contact bach.ipmph2@gmail.com).

## Abbreviations

OHCHR: Office of the High Commissioner for Human Rights
UNFPA: The United Nations Population Fund
UNICEF: United Nations Children’s Fund
UN Women: United Nations Entity for Gender Equality and the Empowerment of Women
